# Radiocaesium in mosses from the Kopački rit Nature Park in Croatia: searching for undeclared releases from nuclear facilities in war-torn Ukraine

**DOI:** 10.2478/aiht-2024-75-3853

**Published:** 2024-06-29

**Authors:** Nora Miljanić, Branimir Zauner, Dinko Babić, Branko Petrinec

**Affiliations:** University of Dubrovnik, Department of Electrical Engineering and Computing, Dubrovnik, Croatia; Institute for Medical Research and Occupational Health, Zagreb, Croatia; Josip Juraj Strossmayer University of Osijek, Faculty of Dental Medicine and Health, Osijek, Croatia

**Keywords:** ^134^Cs, ^137^Cs, airborne radioactive pollution, bioindicator, ^134^Cs, ^137^Cs, bioindikatori, radioaktivno onečišćenje zraka

## Abstract

The invasion of Ukraine and military operations around Ukrainian nuclear power plants and other nuclear facilities have prompted us to search for radiocaesium in mosses from the Kopački Rit Nature Park in Croatia, since mosses are known bioindicators of airborne radioactive pollution, and Kopački Rit is a known low radiocaesium background area. Sampling was finished in August 2023, and our analysis found no elevated radiocaesium levels. Kopački Rit therefore remains a suitable place for future detection of anthropogenic radioactive pollutants.

## Sažetak

Invazija na Ukrajinu i vojne operacije oko ukrajinskih nuklearnih elektrana i drugih nuklearnih postrojenja potaknule su nas da potražimo radiocezij u mahovinama u Parku prirode Kopački rit, jer su mahovine poznati bioindikatori radioaktivnog onečišćenja zraka, a Kopački je rit poznat po niskoj razini onečišćenja radiocezijem. Uzorkovanje je završeno u kolovozu 2023. Naša analiza nije otkrila povišene razine radiocezija. Kopački rit stoga ostaje pogodno mjesto za buduću detekciju antropogenog radioaktivnog onečišćenja.

Due to potentially catastrophic consequences of an accident such as those in Chernobyl and Fukushima power plants, safety regulations related to nuclear technology are very strict, and properly maintained nuclear facilities are ecologically safe, that is, unless their operation is disturbed. In such a case, a release of radioactive material from the facility into the environment may cause an ecological disaster with adverse effects that could last for many decades. For these reasons, the Russian invasion of Ukraine, which began on 24 February 2022, raised a widespread concern that one or more of the four nuclear power plants in Ukraine and some smaller nuclear facilities in the country might become the source of radioactive pollution with long-term consequences.

The beginning of the war in Ukraine basically coincided with the publication of our report (1) on the radioactivity in mosses from the Kopački Rit Nature Park (Kopački rit), which is located in the middle Danube River basin, on the Croatian side of the tripoint of Croatia, Hungary, and Serbia. We found unusually low activity concentrations of radiocaesium in our samples, which implies that very small amounts of these fission-produced radionuclides could be detected in mosses from the Kopački rit. This fact and the position of the Kopački rit relative to Ukraine urged us to repeat moss sampling in April and May 2023 and check if radiocaesium content increased in these bioindicator organisms. We focused on two isotopes of caesium: ^137^Cs (with a half-life of *T*_1/2_=30.1 years) and ^134^Cs (*T*_1/2_=2.1 years). Both radionuclides are abundant products of ^235^U fission, which means that they are anthropogenic. Both are strong emitters of gamma radiation and therefore easily detected and quantified by means of gamma-ray spectrometry, which is a highly sensitive method that requires no sophisticated sample preparation and is consequently both accurate and fast. However, the processes leading to the production of ^137^Cs and ^134^Cs are not exactly the same, which turns out to be advantageous in tracing their origin in the environment. ^134^Cs is formed from stable ^133^Cs by neutron capture, which occurs in a nuclear reactor but not in a nuclear explosion, whereas ^137^Cs is produced in both cases.

Thus, any leakage from a nuclear reactor in Ukraine into the environment would result not only in the appearance of ^134^Cs but also in an increase of overall ^137^Cs concentrations. The detection of such a leakage may depend on various factors: the amount of the released radioactive matter, distance from the source, or atmospheric conditions. While we cannot claim that our results cover all possible scenarios, they still provide a timely insight into the state of the middle Danube River basin, an area that is relatively close to Ukraine, densely populated, and intensely agricultural. Since any appearance of anthropogenic radionuclides in the environment is considered dangerous, not only at the level of food chains but also with regard to all the media and products the humans interact with, studies of this sort are quite important.

## MATERIALS AND METHODS

**Figure 1:**
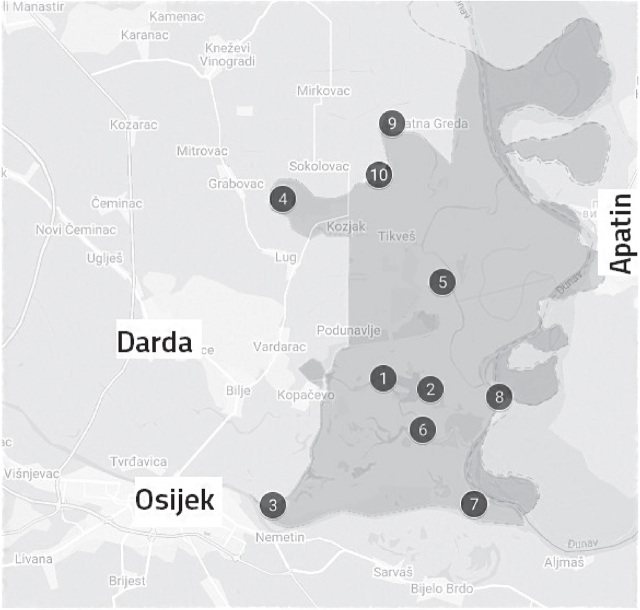
Sampling sites (1–10) within the Kopački Rit Nature Park (shaded)

In our previous study (1) we sampled moss at forty sites across Kopački rit and its vicinity and obtained an in-depth view of the distribution or radiocaesium. For the work presented here, we reduced the number of sites to ten to get an overview of the more recent situation as quickly as possible. The sampling sites were either at Kopački rit or close to its borders (Figure 1). Since site accessibility changes with floods, road conditions, and so on, we could not take samples at the same sites as before.

The experimental procedures were essentially the same as described in our previous study (1). Briefly, in April and May 2023, we again sampled *Anomodon viticulosus*, which is the most abundant moss at Kopački rit. As before, the moss was thoroughly cleaned, dried to constant mass (hence our results refer to dry mass), and packed into 1 L Marinelli beakers. Activity concentrations (*A*) of the samples were determined by means of high-resolution gamma-ray spectrometry, using two high-purity coaxial detectors – an Ortec GMX and an Ortec GEM (Advanced Measurement Technology, Oak Ridge, TN, USA) with respective relative efficiencies of 74.3 % and 57 %, energy resolutions of 2.23 and 1.70 keV, and ^60^Co 1.33 MeV both. ^137^Cs and ^134^Cs activity concentrations were studied at energies of 661.7 and 604.7 keV, respectively. Measurements were carried out 1–2 months after the sampling, and since the half-lives of both radionuclides are much longer, no correction for radioactive decay was required. However, the branching ratio of ^134^Cs for gamma emission at the specified energy was corrected for true coincidence summing (2).

## RESULTS AND DISCUSSION

The distances between Kopački rit and the Ukrainian nuclear power plants (NPPs) are about 780 km for the Khmelnytsky NPP, 820 km for the Rivne NPP, 970 km for the South Ukraine NPP, and 1220 km for the Zaporizhzhia NPP. These are, in fact, small distances for the spread of airborne radionuclides via air masses, having in mind the spread of radioactive matter from the Chernobyl NPP (about 1040 km from Kopački rit) during the 1986 accident (3) or the fact that even the radioactive matter originating from the disaster in Fukushima was detected in Croatia (4). The Chernobyl radioactive spread was not the same in all directions (3), so eastern Croatia received less fallout than many other equally distant areas from the source of radioactive plume. This is evident from generally lower activity concentrations of ^137^Cs in soil in this part of Croatia than elsewhere in the country (5). Our earlier finding (1) that the *A* of ^137^Cs in moss samples from the Kopački rit was unusually low (0.7–13.1 Bq/kg) in 2018 is consistent with that, since the maximum value measured (and extrapolated to 2018) was 65 Bq/kg in Serbia (6), 400 Bq/kg in Greece (7), 300 Bq/kg in Armenia (8), 10,680 Bq/kg in Austria (9), and 360 Bq/kg in Turkey (10). In all these locations, the primary source of measured ^137^Cs was the Chernobyl accident, and the distances between all these countries and Chernobyl are not significantly different.

There are several methods for the detection of airborne radionuclides, each having advantages and disadvantages. The most common is to measure the activity of a filter through which ground-level air has been pumped for some time (5). While this method is the most direct, its disadvantage is that pumping must be carried out while the radioactive matter concentration in the air is high enough, which may not last long (4). Another method is to measure radioactivity concentrations in surface soil, where airborne radionuclides eventually end up. Radionuclides remain in soil for a long time, but their concentrations stabilise only after a long deposition and diffusion. The third approach, which we took in this study, is to use bioindicator organisms which take up airborne radionuclides quickly and retain them for long (11,12,13). This method is particularly suitable for those radionuclides which have similar chemical properties as some biogenic elements, which is the case with caesium. Having the electronic structure similar to those of potassium and sodium, caesium has a potential for mimicking either of these two elements in a tissue.

The results of our measurements are shown in Table 1. We detected ^137^Cs in all samples, the activity concentrations ranging from 1.2 to 5.3 Bq/kg. By comparing these values with the 0.7–13.1 Bq/kg range from our previous study (1), we conclude that that there has been no significant uptake of ^137^Cs by mosses in Kopački rit between August 2018 and May 2023. This implies no contribution of additional ^137^Cs to Kopački rit from any source, including the nuclear facilities in Ukraine. Regarding ^134^Cs, Table 1 shows that activity concentration for each site (sample) is lower than the decision threshold. In other words, no ^134^Cs was detected in Kopački rit in April and May 2023. These clearly suggests that the war in Ukraine has not caused a significant release of radioactive matter from any facility where the fission of ^235^U has been taking place.

**Table 1:** Activity concentrations of ^137^Cs and decision thresholds of ^134^Cs in mosses sampled in the Kopački Rit Nature Park

**Sampling site** (see Figure 1)		**^137^Cs Activity concentration [Bq/kg]**	^134^Cs decision threshold* [Bq/kg]
1	Conakut	5.3±0.3	0.8
2	Kanal Šanta	4.8±0.4	1
3	Bajer	1.2±0.2	0.7
4	Siget	4.6±0.3	1
5	Samaj Siget	3.5±0.4	1
6	Hulovo	3.2±0.3	0.8
7	Renovo	1.5±0.3	1
8	Stari Dunav	2.1±0.6	2
9	Zlatna Greda	2.0±0.4	2
10	KDT	1.9±0.4	1

* ^134^Cs activity concentrations were always below the decision threshold, indicating that there was no ^134^Cs in the measured samples

Therefore, Kopački Rit remains the low radiocaesium background area, suitable for future airborne radionuclide pollutant monitoring.
